# Report on the Specific Nature and Communicability of Erysipelas, and Its Connection with Puerperal Fever, Read to the Cook Co. Medical Society

**Published:** 1852-09

**Authors:** N. S. Davis

**Affiliations:** Prof. Pathology, Practice, and Clinical Medicine, in Rush Med. College; Chicago


THE NORTH-WESTERN
MEDICAL AND SURGICAL JOURNAL.
NEW SERIES.

VOL. I. SEPTEMBER, 1852. No. 5.

### ORIGINAL COMMUNICATIONS.

ART. I.—*Report on the Specific Nature and Communicability of Erysipelas, and its Connection with Puerperal Fever, read to the Cook Co. Medical Society.* By N. S. DAVIS, M. D., and Prof. Pathology, Practice, and Clinical Medicine, in Rush Med. College.

THAT erysipelas is a disease, *sui generis*, that is possessing a specific character, very few will be inclined to doubt. The existence of constitutional disorder antecedent to the development of the local inflammation; the peculiarity of the pain; the tendency to spread, and well defined boundaries of the inflamed part; the little tendency to the effusion of plastic lymph, or the formation of healthy pus; all mark the erysipelatous disease as differing essentially from ordinary inflammation.

In what this difference consists, or on what it depends, is not so apparent. Some have supposed that the peculiar phenomena and results of erysipelas, depended merely on its seat in the cutaneous tissue. If this were true, however, every inflammation of the skin would be erysipelas, which is very far from being the fact.

Others have attributed the peculiarities of this disease wholly to the nature of the existing cause. That the exciting cause may be of such a nature as to determine both the development of inflammation and its specific erysipelatous character, is doubtless true; but that such is not generally the case, I think a little reflection will be sufficient to satisfy every one. For example, it is well known that wounds, bruises, local irritants, exposures to cold, irritating ingesta, &c., are among the common exciting causes of erysipelas; and yet we see these same causes acting every day, even in such intensity as to develope local inflammations, but without imparting to them any of the specific phenomena of the disease under consideration. From the foregoing facts all our more careful observers regard it as necessary to the development of erysipelas, that there should be not only a manifest exciting cause, but a previously existing and peculiar condition of the fluids of the body.

With some persons this peculiar condition appears to be congenital, and they are said to be constitutionally predisposed to erysipelatous attacks; the disease sometimes returning at stated seasons, and in other cases frequently but at irregular intervals of time.

Although most observers regard a previously existing morbid condition of the system as necessary to enable ordinary exciting causes to develope erysipelas, yet few have attempted to define what that condition is.

It does not appear to be accompanied by such a change in any of the solids or fluids as can be detected or measured by chemical agencies. Much light, however, can be thrown on the nature of this condition by carefully comparing the causes that are known to lead to its development with the phenomena of the disease and its effects. With the exception of those remote periods of its epidemic prevalence, erysipelas is a disease very much confined to those who live habitually in filthy and ill-ventilated apartments, or who are addicted to intemperate habits. It is well known that the narrow and filthy alleys of our cities, and the crowded apartments of hospitals, jails, poor-houses, &c., furnish far the larger number of victims to this disease. In this respect, as well as in many of its active phenomena, the disease bears a close relation to typhus and malignant dysentery.

No fact is more susceptible of proof, than that the impure air of crowded apartments impairs the elementary property of tonicity in all the solids, and so changes the quality of the blood, that it does not maintain a due degree of nervous energy or a healthy state of nutrition. A state not identical but closely parallel, is produced by the excessive use of alcoholic beverages. While the first effect of these liquors is exhilerating to the nervous system, they very decidedly diminish the organic actions, and thereby allow carbon to accumulate until it impairs the quality of the blood and the tonicity of the solids to a degree but little less than that induced by the impure and contaminated air of crowded jails, &c. If we connect with these circumstances the essentially typhoid tendency of the fever accompanying severe erysipelas, and the imperfect character of the suppuration which sometimes results, (the latter being more like a dissolution of the cellular tissue than a proper process of suppuration), we shall see clearly that the essential pre-existing condition, to which authors allude, as necessary to the development of erysipelatous inflammation, is one of impaired tonicity in the solids, and of diminished healthy stimulating qualities in the fluids. The degree of change thus previously induced will determine the degree of malignancy or typhoid character of the disease. If tonicity be but little impaired and the immediate exciting cause one calculated to develope a disease of excitement, the constitutional symptoms accompanying the erysipelas will be sthenic, or more purely inflammatory in their character. Such is frequently the case in sporadic cases occurring in country districts, while the opposite is almost always found in crowded cities and public institutions. While all seem to admit the frequent spontaneous origin of erysipelas, and its occasional prevalence as an epidemic depending on some atmospheric or unknown cause, much diversity of opinion prevails in regard to the question whether it is, under any circumstances, contagious or communicable from one individual to another. On this point we find the same variety of conflicting views and apparently contradictory facts, as exists in reference to the contagiousness of yellow fever, dysentery, cholera, &c. On the one hand, cases are detailed with much circumspection in which it would seem that the disease had an unmistakable origin from contact with those previously affected with it. Thus the introduction of a severe case of erysipelas into the wards of a hospital has been speedily followed by other cases among the inmates who were previously unaffected by it. So, too, individual members of a family have been known to be attacked successively with such a degree of regularity as to impress strongly the idea that they had taken it one from another. Such an instance recently came under my own observation. On the other hand, we have repeatedly had cases of this disease in the large and well ventilated wards of the Illinois General Hospital, in the midst of beds containing patients laboring under a variety of other diseases, when neither they nor the attendants suffered the slightest symptoms of an attack. And in the family to which I just alluded, where three persons, (the mother and two daughters) were attacked in succession, they had all been exposed to the same general causes, and were all in the habit of sleeping in a chamber much too small and low to furnish a sufficient quantity of pure air for the number lodging in it. And it should be added that none of the neighbors or others, who visited them during their sickness, took the disease. So far as my own personal observations go, they fully coincide with the experience of Dr. Wood, as expressed in his recent valuable work on Practice, in which he states, that neither in hospital nor private practice has he seen any decided evidence of the communication of erysipelas by direct *contagion.*

In making this statement, I wish the members of the society to keep in mind the plain distinction between contagion and infection. The first I restrict entirely to a morbid agent or poison generated in the body of the sick, and capable of inducing the same disease by being introduced into the system of another person previously in good health. By *infection*, I mean such a morbid or impure state of the atmosphere in any given room or locality as will render healthy persons who come in contact with it, liable to attacks of whatever disease may be prevalent at the time; such morbid or infectious condition of the atmosphere not being produced by any specific poison generated in the body of the sick, but either by insufficient ventilation and cleanliness acting in connection with an accumulation of ordinary animal excretions, as is often seen on emigrant ships, in jails, hospitals, narrow and neglected alleys and cellars in cities, &c.; or by some inscrutable change giving rise to the epidemic prevalence of particular diseases. In this sense I have no doubt but erysipelas has often become highly *infectious,* especially in camps, jails, and other confined and filthy localities. The disease once begun in such a place is very liable to spread, and to attack healthy persons who may chance to come much within the infected locality. And not unfrequently persons only temporarily exposed will sicken after they have returned to a remote and perhaps healthy place. Yet any number of patients sick with the disease may be transferred to a dry, pure, and healthy region and the utmost freedom of contact will fail to propagate the disease. I think if all medical observers and writers would make the clear distinction, here stated, between *contagion* and *infection*, we should have an end of the long protracted controversy about the contagiousness of erysipelas, cholera, typhus fever, and other kindred affections.

Having dwelt thus long on the nature and communicability of erysipelas, my remarks on its connection with puerperal fever must be very brief.

At a comparatively early period in the history of our profession, it was observed that whenever erysipelas prevailed, either as an epidemic or in the crowded wards of a hospital, women in child-bed were much more liable than usual to attacks of puerperal fever, or uterine and peritoneal inflammation. And the coincidence has been noticed by all the more recent observers.

Several of those physicians who published accounts of the epidemic erysipelas that prevailed in many parts of our country in 1842–3–4, noticed in connection therewith the prevalence of puerperal fever at the same time and in the same localities. Some of the most eminent obstetrical practitioners in Europe have alleged a still closer relation between the two diseases, and have recorded cases seeming to show that a practitioner in attending on a case of severe erysipelas was likely, without great precautions, to communicate the disease, in the form of puerperal fever, to those patients whom he might attend in child-bed. Thus alleging not only a direct connection but a mutual communicability of the two diseases. In further proof of the same position we find many cases recorded in which the puerperal disease attacked the patients of a single practitioner with such regularity as to strongly impress the idea that the cause of the disease was communicated from one to another by the practitioner himself. Indeed, so pertinaciously has it been known to adhere to the pathway of a single practitioner, that no amount of precaution in regard to cleanliness, change of clothing, &c., would apparently prevent his communicating it to others, and in one instance, at least, it is said that after the unfortunate practitioner had retired wholly from his patients for several weeks, the first woman he attended in confinement after his return was attacked with the uterine and peritoneal inflammation. These facts, however, as has been well remarked by another, prove too much. And instead of sustaining the doctrine of contagion or direct communicability, they would go far to satisfy us that there were other and coincident causes at work at the same time. For surely that must be a remarkably tenacious poison that would adhere to the fingers of a practitioner through three or four weeks of daily ablutions, changes of air, clothing, &c., in a degree of activity sufficient to develope a virulent disease in the first puerperal case with which he comes in contact. In my own experience of fifteen years both in city and country practice, I have been so fortunate as to see no cases of even seemingly communicated cases of either erysipelas or puerperal fever. And, after a somewhat careful search, I have found no such cases on record except those that occurred at the time and place, when one disease or the other was prevailing as an epidemic.

This is a fact which does not appear to have been kept sufficiently in mind, while discussing this subject.

We can very readily conceive how the same circumstances, and the same conditions of the system, that have been already described as favorable and necessary to the development of erysipelas, should likewise readily give rise to uterine and peritoneal disease during child-bed; and thereby give the two diseases a coincident prevalence without any direct or even indirect communication. Still I would by no means excuse the practitioner from using the utmost precautions against bringing erysipelatous cases in contact, even in the most indirect manner, with his puerperal cases. The most urgent calls on my time and attention have prevented me from giving you at this meeting anything beyond this hasty glance at the topics on which I was required to report.

CHICAGO, *August* 31, 1852.

